# An Evaluation of the Subcutaneous Depot Release of TV-46000, A Novel Long-Acting Injectable (LAI) Formulation of Risperidone, Under Extreme Conditions in Dogs, Minipigs and Humans †

**DOI:** 10.3390/pharmaceutics17020150

**Published:** 2025-01-22

**Authors:** Lilach Steiner, David Bibi, Avia Merenlender Wagner, Pavel Farkas, Safra Rudnick-Glick, Pippa Loupe, Hussein Hallak

**Affiliations:** Innovative Research and Development, Teva Pharmaceutical Industries, Ltd., Netanya 420483, Israel

**Keywords:** risperidone, TV-46000, schizophrenia, long-acting injectable, antipsychotic, drug delivery depot technology

## Abstract

Background: TV-46000 (Uzedy, Teva), a long-acting subcutaneous antipsychotic, is an injectable formulation of risperidone and is approved by the FDA for the treatment of schizophrenia in adults. Its innovative copolymer-based drug delivery depot technology (licensed from MedinCell, Jacou, France) allows for plasma concentrations of the total active moiety of risperidone (TAM) to reach clinically relevant levels within 6–24 h and the maintenance of these therapeutic levels with monthly and bimonthly dosing regimens. Objective: As part of the development program for TV-46000, the effect of extrinsic factors of manipulation on the site of injection, and on the pharmacokinetic (PK) profile of TAM following TV-46000 administration was evaluated. Methods: Studies were conducted assessing the effect of heat and rubbing with male Gottingen minipigs and the effect of rubbing with male beagle dogs. A pilot clinical study in healthy volunteers was performed to evaluate the effect of rubbing. Results: These investigations showed that heating or rubbing of the TV-46000 sc injection site immediately post-injection had no clinically meaningful impact on safety and no burst or uncontrolled release was evident. Furthermore, no impact of injection site manipulation on TAM exposure was observed after depot formation (≥0.5 h post-injection). Conclusions: The observed similarity in findings between the animal and human studies supports the suitability of animal models for evaluation of the effect of extrinsic factors on injection sites and its translatability to clinical settings.

## 1. Introduction

Risperidone is a second-generation antipsychotic medication widely used in the treatment of schizophrenia, bipolar I disorder and autism-associated irritability [1,2,3,4]. Risperidone has been well characterized and is currently available as oral, intramuscular (im) or subcutaneous (sc) injectable formulations. Both the parent drug and the main metabolite, 9-hydroxyrisperidone (9-OH risperidone), are active, and act as antagonists of dopamine-2 and serotonin-2 receptors [2,5,6]. Although risperidone has been shown to be highly effective as an antipsychotic treatment, high relapse rates due to suboptimal adherence to medication regimen significantly impact patients’ functional decline [7,8]. Thus, to prevent relapse and maintain functional stability, long-term uninterrupted treatment is beneficial. The utilization of a LAI antipsychotic agent may enhance adherence and compliance in patients diagnosed with schizophrenia [9]. Furthermore, neuroimaging evidence suggests that early use of LAI risperidone could lead to improved clinical outcomes in these patients [10].

Long-acting injectable (LAI) or depot delivery systems refer to formulations designed for prolonged and sustained drug release over an extended period, which can range from a few weeks to several months, thus reducing dosing frequency and simplifying the drug regimen [11,12]. Various technologies are available for LAI systems, such as oil-based formulations, polymer implants or drug suspensions. All currently available antipsychotic LAIs are administered either via the im or sc route [13,14]. Following LAI administration, a depot is formed at the injection site, serving as a reservoir from which the drug is constantly released. First, there is an initial release of the drug after the LAI injection, followed by continuous drug release. The gradual absorption process contributes to maintaining stable concentration of the drug in the bloodstream.

TV-46000 (Uzedy, Teva) is a long-acting sc antipsychotic (LASCA) and was approved by the FDA in April 2023 as an extended-release injectable risperidone treatment for schizophrenia in adults. In a phase 3 study, the RISE study, TV-46000 administered once monthly (q1m) or once every 2 months (q2m) was found to significantly delay time to relapse and decrease the risk of relapse in comparison with placebo [15]. In phase 1 and 3 clinical studies, TV-46000 was shown to have a safety profile similar to that of other risperidone formulations with a minimally invasive route of administration via sc injection [15,16,17].

TV-46000′s innovative copolymer-based drug delivery depot technology (licensed from MedinCell, Jacou, France) allows for plasma concentrations of the total active moiety (sum of risperidone and 9-OH risperidone; TAM) to reach clinically relevant levels within 6–24 h from administration without a loading dose or oral supplementation and the maintenance of these therapeutic levels with monthly and bimonthly TV-46000 sc injections [16]. The depot technology consists of methoxy-poly(ethylene glycol)-co-poly(D,L-lactide) (15% *w*/*w*) and poly(D,L-lactide)-co-poly(ethylene glycol)-copoly(D,L-lactide) (10% *w*/*w*) polymers, and dimethyl sulfoxide (45% *w*/*w*), which regulate the rate and duration of drug release [18].

Studies have demonstrated that extrinsic factors can influence drug release and the systemic absorption of LAI products following sc administration. Mechanical stimulation, including rubbing or applying heat to the injection site, may enhance drug movement through increased blood flow and capillary permeability. These actions could alter the PK profile of a drug, potentially impacting drug absorption and distribution [1,19,20,21]. During the development program for LAI products, regulatory agencies have raised requests and considerations regarding these potential variations, highlighting the need for the careful evaluation of factors that might affect drug delivery mechanisms. Thus, as previously described in a conference presentation at AAPS 2023 [22], the effect of the manipulation of the injection site on TV-46000 sc depot formation and stability was assessed as part of the development program for TV-46000 by evaluating the effect of extrinsic factors applied to the site of injection at different timepoints post-injection on the PK profile of TAM. Non-clinical studies evaluating the effect of heating and rubbing at the site of injection were conducted using male Gottingen minipigs and beagle dogs. The minipigs and dogs were selected as the non-rodent species due to their acceptance by various regulatory authorities for evaluating local tolerability following subcutaneous injection. Additionally, the metabolic profile of risperidone in these animals is similar to that in humans (*data on file*, Teva Pharmaceuticals). To bridge the extensive assessment of the effect of extrinsic factors on TV-46000’s PK profile in animals to a clinical setting in humans, an early pilot phase 1 study of healthy volunteers was conducted in which the effect of extreme rubbing of the injection site was investigated.

## 2. Materials and Methods

### 2.1. Heating Evaluation of TV-46000 Following SC Administration in Male Göttingen Minipigs

The effect of heating the injection site on the PK profile, systemic toxicity and tolerance of TV-46000 following a single sc injection in minipigs was investigated in this study. For the study, two groups of male minipigs (n = 8/group), were given single sc injections of TV-46000, a 50 mg dose on the dorsum right side of the spine. A 50 mg dose of TV-46000 corresponds to the lowest clinical dose, mimicking real-world scenarios. This dose was chosen for its sensitivity, as the smaller dose volume (test item at a concentration of 360 mg/mL, at a dose volume of 0.14 mL/site, to give a dosage of 50 mg/minipig), based on the surface area-to-volume ratio, allows for better evaluation of extrinsic factors. 

Group 1 was subjected to consecutive 20 min heating (40–42 °C) sessions of the injected site at 2, 24, 72, 120 and 168 h post-dose. For the heating application, a stainless-steel heating-convey pipe (5 cm radius), connected with an infrared light source, was directed to the exposed injection site and placed at a predetermined heating distance. A probe, connected with a digital thermometer, was placed at the surface of the injection site. The probe was inserted under a protective leather cover, and out through an open-holed injection site, to record the temperature of the site, which was maintained at 40–42 °C. Group 2 animals received a TV-46000 injection with no heating of the injection site. The PK of risperidone, 9-OH risperidone and TAM were evaluated in the presence or absence of heating. For the PK evaluation, blood samples of 1 mL each were collected into EDTA tubes from the cannulated right external jugular vein of all animals, at the following time points: pre-dose, 0.5, 1 and 2 h post-injection and before the initiation of each heating treatment, and then 0.5, 1, 3 and 6 h after the end of each heating session. Additionally, all animals were examined for clinical signs twice on Day 1 and once daily thereafter up to necropsy. Treatment sites were observed for the evaluation of reaction to injection (induration and mechanical swelling), pain at palpation, the presence of a depot and inflammation (erythema, cutaneous heat, swelling, hardening and itching) from Day 1 up to necropsy (Day 30).

### 2.2. Rubbing Evaluation Following Administration of TV-46000 in Male Göttingen Minipigs

The effect of local rubbing of the injection site on the PK profile, systemic toxicity and tolerance of a single sc injection of TV-46000 (50 mg/pig) was investigated in three groups of male minipigs (n = 3 or 4 per group).

In Group 1, the rubbing of the injection site was applied to animals at 1, 2, 3 and 6 h post-TV-46000 injection and in Group 2 at 24, 48 and 72 h post-injection. The rubbing was applied using the lateral side of a 10 mL syringe, rolling over the marked injection site for a 1 min duration with at least 30 back-and-forth rolls. In Group 3, no rubbing was performed on animals. All animals were observed for 32 days.

For PK evaluation, blood samples of 1 mL each were collected into EDTA tubes from each group at the time points indicated below.

Group 1: pre-dose, 1*, 1.5, 2*, 2.5, 3*, 3.5, 6*, 6.5, 24, 48, 72, 168, 360 and 720 h post-dose.

Group 2: pre-dose, 1, 3, 6, 24*, 24.5, 48*, 48.5, 72*, 72.5, 168, 360 and 720 h post-dose.

Group 3: pre-dose, 1, 1.5, 2, 2.5, 3, 3.5, 6, 6.5, 24, 24.5, 48, 48.5, 72, 72.5, 168, 360 and 720 h post dose.

* means timepoints of rubbing of injection site; at these times, blood samples were collected prior to massage of the injection site.

Additionally, all animals were examined for clinical signs at least 3 times on Day 1 and once daily thereafter up to necropsy on Day 33. Treatment sites were observed for the evaluation of reaction to injection (induration and mechanical swelling), pain at palpation, the presence of a depot and inflammation (erythema, cutaneous heat, swelling, hardening and itching) from Day 1 up to Day 33.

### 2.3. Rubbing Evaluation Following Administration of TV-46000 in Male Beagle Dogs

TV-46000 was administered to five groups of male beagle dogs as a single sc dose of 12.5 mg (35 µL/dog). This study was conducted after the pilot clinical study described in Section 2.4 to replicate its design and facilitate bridging between animals and humans, using a 12.5 mg dose. This dose, being relatively low, was expected to exhibit an exaggerated response to extrinsic factors due to the enhanced surface area-to-volume ratio, which makes the depot more susceptible. The control group, Group 1, did not receive a rubbing at the site of TV-46000 injection. For the other groups, the injection site was rubbed by applying moderate pressure in a back-and-forth motion with the palmar surface of the gloved fingers by the administrator, for 30 s (±5 s). The same administrator rubbed the injection site for all animals to ensure the procedure was standardized. For each group, rubbing the injection site was performed at different time points: immediately after injection (Group 2), 30 min (Group 3), 1 h (Group 4) and 4 h (Group 5) post-injection. For PK evaluation, 2 mL blood samples were collected into plastic vials containing EDTA as anticoagulant at the following time points after TV-46000 injection: pre-dose, 0.5, 1, 1.5, 2, 3, 4, 4.5, 5, 8, 12, 24, 48, 72, 120,168, 240, 288, 360, 480, 576 and 672 h post-dose. Additionally, all animals were examined for clinical signs during the acclimatization period (entrance examination and before randomization) then once a week thereafter up to necropsy. Treatment sites were observed for the evaluation of reaction to injection (induration and mechanical swelling), pain at palpation, the presence of a depot and inflammation (erythema, cutaneous heat, swelling, hardening and itching) from Day 1 to Day 30 (end of the study).

### 2.4. Phase 1 Clinical Study in Healthy Volunteers

In a phase 1 clinical study in healthy volunteers (the RISPE1ZG15EU study), 10 participants were administered a single sc injection of TV-46000 at a dose of 12.5 mg (35 µL injection volume) and received a rubbing at the injection site immediately after injection. The injection site was rubbed by applying moderate pressure in a back-and-forth motion using the palmar surface of gloved fingers by the same administrator for 30 s (±5 s) to ensure the procedure was standardized. A second group of participants (n = 9) received the same single sc injection of TV-46000 at a dose of 12.5 mg without a massage at the injection site.

The safety parameters included adverse event reporting, ECG, laboratory measures, physical exams and injection site examinations. Blood samples for the determination of risperidone and 9-OH-risperidone plasma concentrations were collected at prespecified time points pre-dose and throughout 54 days following TV-46000 dosing. Specifically, samples were collected at the following times: immediately before the TV-46000 injection and 0.5, 1, 2, 3, 4, 6, 8, 12, 22, 24, 26, 30, 36, 48, and 72 h post-dose; and thereafter once 24 h for 9 days and then every 6 days until 54 days post-TV-46000 dosing.

### 2.5. Bioanalytical Methods

During the conducting of the non-clinical and clinical program for TV-46000, all concentrations of risperidone and 9-OH-risperidone in plasma were quantitated using liquid chromatography with tandem mass spectrometry (LC-MS/MS) detection bioanalytical methods that had been validated with respect to sensitivity, specificity, accuracy, precision and stability in accordance with bioanalytical method validation guidelines [23,24].

### 2.6. Pharmacokinetic Analysis

PK samples were collected at each time point to generate PK profile curves of risperidone, 9-OH risperidone and TAM concentrations versus time. For calculation of the mean concentrations versus time curves, the concentrations below the quantification limit (LOQ) were accepted as zero. The LOQ values were 0.1 ng/mL for risperidone and 9-OH risperidone for the minipig and dog studies, and 22.2 pg/mL and 19.7 pg/mL for risperidone and 9-OH risperidone concentrations in the human study. TAM concentrations for all animals were derived for each timepoint post-injection of TV-46000. These individual concentrations within a group were averaged at each time point to generate the mean concentrations versus time curves for each group. PK analysis was performed using Kinetica^TM^, version 4.4.1 (Thermo Electron Corporation, Waltham, MA, USA) for minipig studies and Phoenix WinNonlin^®^ software version 6.3 (Pharsight Corporation, Princeton NJ, USA) for dog and human studies. Individual and mean plasma concentrations versus time curves were evaluated using the non-compartmental method. For calculations, the nominal sampling times were used. The apparent terminal elimination half-life, AUC_0–∞_ and related parameters were accepted only if the terminal phase fit was good (Rsq adj value ≥ 0.85) and/or the extrapolated part of the curve (AUC_ext%_) was lower than 20%. Otherwise, the terminal half-life and half-life-related parameters were not reported. For calculation of the dose-normalized parameters of 9-OH-risperidone and total active moiety, the actual dose of the parent drug, TV-46000, was used.

## 3. Results

### 3.1. Effect of Heating of Injection Site Following Administration of TV-46000 in Male Göttingen Minipigs

**Figure 1:**
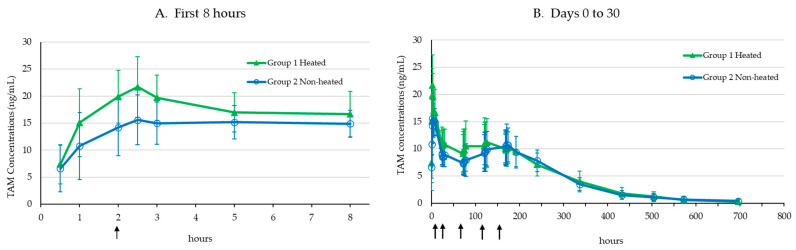
Mean (±SD) TAM concentrations before and after heat application (shown as arrows) on the site of injection at 2 h, 1, 3, 5 and 7 days post-50 mg sc TV-46000 dose, for the first 8 h (**A**) and on Days 0 to 30 (**B**).

Following a sc administration of TV-46000 in male Göttingen minipigs, risperidone and 9-OH risperidone concentrations were found in the plasma from the first collection timepoint, 30 min after administration up to the last collection timepoint on Day 30 of the study in all animals. As shown in Figure 1, mean TAM plasma concentrations were similar over time in Groups 1 and 2, indicating that the heating of the treatment site did not affect the distribution of the substance. From Day 9 (192 h), plasma levels gradually decreased to achieve comparable values on Day 30 (720 h).

Mean PK parameters (±SD) following TV-46000 sc injections for risperidone, 9-OH risperidone and TAM are presented in Table 1. No substantial differences were seen in the overall TAM exposure (AUC_0-tlast_) between the heated and non-heated treatment groups (AUC_0-tlast_ of 3514.6 ng × h/mL and 3239.9 ng × h/mL, respectively).

Slight swelling and/or induration of the injection site, due to the presence of a depot, was observed in the majority of animals from both groups. The incidence was similar in both groups, and was resolved by Day 30.

### 3.2. Effect of Rubbing of Injection Site Following Administration of TV-46000 in Male Gottingen Minipigs

**Figure 2:**
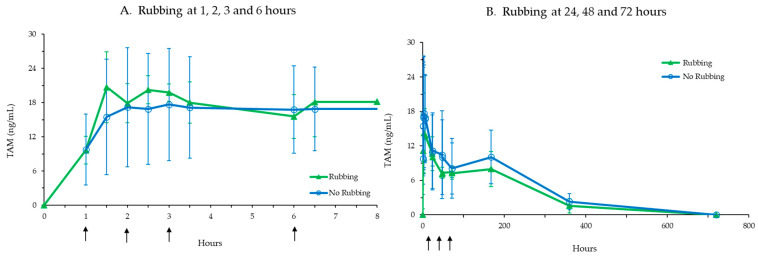
TAM concentrations following injection site rubbing (shown as arrows) of the Group 1 minipigs (n = 3) at 1 h, 2 h, 3 h and 6 h post-50 mg dose (**A**) and Group 2 (n = 3) at 24, 48 and 72 h post-50 mg dose (**B**).

Following sc injection of TV-46000, risperidone and 9-OH risperidone concentrations were detected starting with the first PK sample at 1 h post-injection to 360 h post-injection in all animals. Concentrations were below the LOQ of 0.1 ng/mL for most of the animals at the next sampling time at 720 h, with the exception of two animals, in which risperidone and 9-OH risperidone were measured at 720 and 888 h post-injection. As shown in Table 2, the T_max_ for risperidone occurred between 1 and 6 h post-dose, while for 9-OH risperidone, T_max_ generally occurred between 6 and 6.5 h post-dose. No differences between treatment groups due to rubbing of the injection site were seen in overall TAM exposure (AUC_0-tlast_). Importantly, no clear differences were seen in the TAM plasma concentrations following the massage time points (1, 2, 3, and 6 h post-injection, and again at 24, 48 and 72 h post 50 mg dose, as shown in Figure 2), compared to animals not receiving any massage. This was also shown by the similar TAM exposure values (C_max_ and AUC_0-tlast_) between the groups, suggesting that rubbing the injection site did not have any effects on the release of risperidone. TV-46000 sc injections were well tolerated in minipigs and rubbing did not induce any local intolerance at the injection site.

### 3.3. Effect of Rubbing of Injection Site Following Administration of TV-46000 in Beagle Dogs

**Figure 3:**
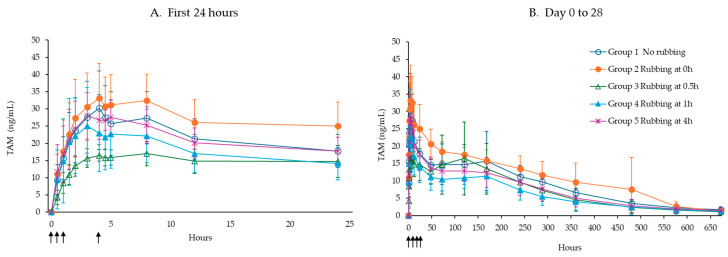
Mean (±SD) TAM concentration following a single TV-46000 injection in Group 1 without rubbing of the injection site and Groups 2–5 with rubbing of the injection site (immediately, at 0.5, 1 and 4 h, shown as arrows) during the first 24 h (**A**) and from Days 0 to 28 post-dose (**B**).

Rubbing the injection site immediately post-injection (Group 2, 0 h) resulted in consistent increases in C_max/dose_ (123%) and total exposure, AUC_last/dose_ (133%) of TAM (Table 3). In agreement with the study in minipigs, rubbing at different times post-injection did not cause increases in risperidone, 9-OH risperidone or TAM exposure (illustrated for TAM concentrations in Figure 3). The release characteristics of TV-46000 were not found to be altered due to rubbing at the injection site as reflected by similar half-life values across the different group (Supplemental Table S1). In general, the animals tolerated sc injections of TV-46000 and rubbing did not induce any local intolerance at the injection site.

### 3.4. Effect of Rubbing of Injection Following TV-46000 Administration in Healthy Volunteers

**Figure 4:**
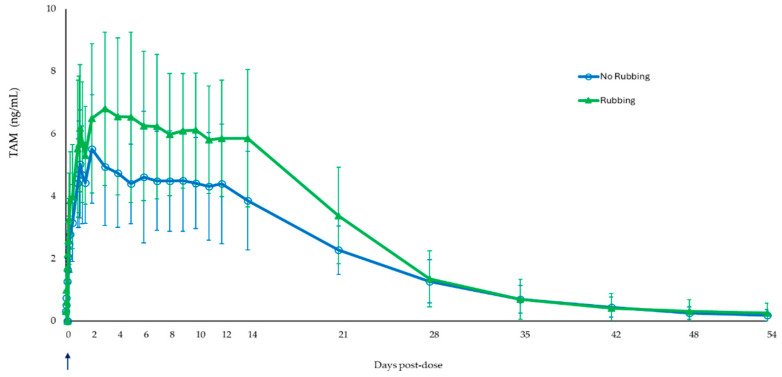
Mean (±SD) TAM plasma concentrations following rubbing of the site of injection immediately (shown as arrow) post-dose in humans.

As observed in dogs, there was only a slight increase in peak exposure of TAM as measured by C_max_ (23% increase) following the extreme challenge of rubbing the injection site immediately after sc injection (Table 4). A 34% and 35% increase in TAM exposure, AUC_0-t_ and AUC_0–∞_, respectively, were observed following administration of TV-46000 with immediate rubbing of the injection site. The release characteristics of TV-46000 were not found to be altered as a result of the intense rubbing as reflected by the geometric mean t_½_ values of 8 days for both groups (194 h versus 199 h for rubbing and no rubbing post-dose groups, respectively) (Figure 4). Overall, results suggested the robustness and extended-release characteristic of the formulation over time did not change following rubbing immediately post sc injection, and the depot still formed very effectively when subjected to this extreme challenge.

In regard to safety measures, there were 8 (89%) reports of adverse events in the group with no rubbing treatment and 4 (40%) in the group that received rubbing of the injection site. Clinical laboratory tests, ECG, vital signs and physical examination did not reveal any clinically meaningful trends in mean changes from baseline or clinically relevant findings in any of these assessments.

## 4. Discussion

TV-46000 sc depot formation and stability were tested in vivo by applying heat and rubbing to the drug injection site at several time points post-dose, and monitoring safety and PK profiles in three pre-clinical studies and one clinical study of healthy volunteers.

In all studies, heating or rubbing of the injection site at all the tested times post-injection was well tolerated and no local adverse reactions were observed. In minipig studies, the release of risperidone and its related PK profile were not affected by heating 2 to 168 h post sc injection, nor by rubbing 1 to 72 h post-dose. TAM peak concentrations were observed at 2 to 3 h post-injection for all groups, regardless of heating or rubbing sessions. TAM plasma concentrations sampled in animals after each heating or rubbing session demonstrated similar levels to those of animals not treated with heating or rubbing of the injection site. Moreover, a similar concentration–time profile was demonstrated for all treatment groups, indicating that neither heating nor rubbing affected risperidone’s release, with it retaining a similar release profile and similar exposure.

In dogs, immediate rubbing of the site of injection still allowed the depot to form and control the release of the drug with only a slight increase in the exposure of TAM. This slight increase in exposure in the group where rubbing was applied immediately after injection appears to be consistent with results obtained in the pilot clinical study, in which rubbing immediately post-injection resulted in similar increases in exposure. Furthermore, the impact of manipulation of the injection site in humans, like in the animal studies, had no clinically meaningful impact on the safety profile and no burst or uncontrolled release was observed. Although some increase in exposure was evident in the rubbing cohort in humans, the depot still formed effectively when subjected to this extreme challenge. When considering TAM exposures, the bioavailability comparison results indicated that rubbing of the injection site immediately after sc administration resulted in a 23% increase in C_max_ and a 35% increase in the AUC_0–∞_ of TAM. Despite these changes, TV-46000’s extended-release characteristics and a multiphasic elimination slope were maintained following this extreme challenge, and no burst or uncontrolled release from the depot was evident. Furthermore, there was no evidence of dose dumping in the clinical studies conducted with TV-46000 [16]. Importantly, in all studies, TV-46000 was locally and systemically tolerated with no substantial differences in local or systemic effects between control and treatment groups [15,16,17].

These findings highlight the robustness of the TV-46000 formulation under real-world conditions. The results suggest that TV-46000 maintains its extended-release characteristics even when subjected to inadvertent manipulation, such as mild trauma or rubbing shortly after administration. This robustness minimizes potential concerns regarding uncontrolled drug release or safety issues, supporting the product’s suitability for clinical use. While the pre-clinical and clinical studies provide strong evidence of formulation stability, it is important to acknowledge that extreme or repeated manipulation beyond those tested was not evaluated. Additionally, potential interspecies differences in skin structure and drug absorption, while considered in the study design, may limit the direct translation of animal model results to all human populations. However, the consistency of findings across animal models and humans strengthens confidence in the product’s performance.

## 5. Conclusions

Following TV-46000 sc injection, a depot was formed enabling the release of risperidone in a controlled manner. Both rubbing or heating of the injection site did not interfere with the formation of the depot and the release of the drug. The observed similarity between minipig, dog and human rubbing results supports the translatability from animal models to clinical settings with regard to exploring the effects of extrinsic factors.

## Figures and Tables

**Table 1:** Mean (±SD) PK parameters following TV-46000 sc injections for risperidone, 9-OH risperidone and TAM for the heated and non-heated groups.

	Group 1 Heated (n = 8)			Group 2 Non-Heated (n = 8)		
	Risperidone	9-OH risperidone	TAM	Risperidone	9-OH risperidone	TAM
C_max_ (ng/mL)	16.3 (4.4)	8.6 (1.5)	21.9 (5.6)	12.6 (4.0)	6.2 (1.4)	16.6 (3.8)
T_max_ (h) ^a^	2 (1–2.5)	7.0 (2.5–192)	2.5 (2.5–5.5)	2.3 (1–8.5)	8.5 (5.5–192)	4.5 (1–8.5)
T_1/2_ (h)	92.3 (33.0)	94.7 (35.9)	92.4 (33.8)	126.6 (44.5)	111.0 (40.0)	121.5 (42.7)
AUC_0-tlast_ (ng × h/mL)	1687.7 (128.4)	1822.4 (404.9)	3514.6 (524.6)	1680.7 (354.1)	1559.2 (356.7)	3239.9 (674.5)
AUC_0–∞_ (ng × h/mL)	1720.3 (121.8)	1864.7 (407.3)	3603.1 (562.1)	1730.3 (334.2)	1602.6 (345.6)	3302.7 (302.0)

^a^ median (min–max range).

**Table 2:** Mean PK parameters (±SD) following TV-46000 sc injections for risperidone, 9-OH risperidone and TAM for groups receiving rubbing at the injection site at different timepoints vs non-massage controls.

	Group 1 Rubbing 1,2,3, and 6 h Post-Dose (n = 3)			Group 2 Rubbing 24, 48 and 72 h Post-Dose (n = 3)			Group 3 No Rubbing (n = 4)		
	Risperidone	9-OH risperidone	TAM	Risperidone	9-OH risperidone	TAM	Risperidone	9-OH risperidone	TAM
C_max_ (ng/mL)	18.5 (6.1)	7.5 (2.1)	23.6 (4.9)	11.6 (6.9)	6.7 (2.7)	15.4 (6.5)	14.1 (7.8) 55.7%	7.8 (4.0) 51.2%	18.9 (9.5)
T_max_ (h) ^a^	2.5 (1.5–2.5)	6.5 (6.5–24)	3.0 (1.5–6.5)	3.0 (1–6)	6.0 (6–24.5)	3.0 (1–168)	1.8 (1.56)	6.5 (6.5–6.5)	3.3 (2–6.5)
T_1/2_ (h)	87.7 (30.6)	101.2 (44.4)	96.7 (29.4)	103.7 (30.8)	117.7 (34.6)	112.0 (31.1)	145.5 (44.8)	143.5 (43.4)	144.0 (43.9)
AUC_0-tlast_ (ng × h/mL)	1294.7 (223.1)	1585.6 (345.3)	2880.3 (556.9)	1204.9 (430.5) 35.7%	1323.8 (240.7)	2528.7 (667.8)	1329.4 (474.3)	1822.9 (749.8)	3152.3 (1222.5)
AUC_0–360h_ (ng × h/mL)	1294.7 (223.1)	1585.6 (345.3)	2880.3 (556.9)	1114.6 (330.4) 29.6%	1209.1 (119.7)	2323.7 (441.8)	1217.3 (528.0)	1653.2 (802.1)	2870.6 (1328.4)
AUC_0–∞_ (ng × h/mL)	1353.7 (171.9)	1683.0 (291.0)	3033.1 (451.7)	1255.5 (455.0)	1408.2 (258.6)	2663.2 (712.8)	1443.9 (465.4)	1973.2 (781.2)	3407.9 (1228.9)

^a^ median (min–max range).

**Table 3:** Relative bioavailability of TAM concentrations in relation to the time of rubbing at the site of injection.

Relative TAM Exposure/Rubbing Time (h)	Group 1 No Rubbing (n = 6)	Group 2 Immediately 0.0 h Post-Dose (n = 6)	Group 3 0.5 h Post-Dose (n = 6)	Group 4 1.0 h Post-Dose (n = 6)	Group 5 4.0 h Post-Dose (n = 6)
C_max/dose_ ^a^	100	123	84.5	97.0	120 ^b^
AUC_last/dose_ ^b^	100	133	103	81.7	99.4

^a^ Relative TAM exposure calculated as: (C_max/dose_GroupX_/C_max/dose_Group1_) × 100. ^b^ Relative TAM exposure calculated as (AUC_last/dose_GroupX_/AUC_last/dose_Group1_) × 100.

**Table 4:** Relative bioavailability of TAM concentrations in relation to immediate rubbing vs. no rubbing following a single sc injection of TV-46000.

Relative TAM Exposure	Test Group Rubbing Immediately After Injection Geometric Mean (%CV) (n = 10)	Reference Group No Rubbing Geometric Mean (%CV) (n = 9)	Test/Reference Ratio (%)
C_max_ (ng/mL)	7.4 (33.3)	6.0 (30.4)	123.0
AUC_0-t_ (ng × h/mL)	3440 (27.1)	2570 (25.6)	133.9
AUC_0–∞_ (ng × h/mL)	3590 (25.8)	2660 (25.6)	135.0

## Data Availability

The data sets used and/or analyzed for the studies described in this manuscript are available upon reasonable request.

## Supplementary Materials

The following supporting information can be downloaded at: https://www.mdpi.com/article/10.3390/pharmaceutics17020150/s1, Table S1. Mean PK parameters (±SD) following TV-46000 sc injections for TAM concentrations in male Beagle dogs following rubbing at the injection site at different timepoints vs non-rubbing controls.
