# Supplementary material for: An Evaluation of the Subcutaneous Depot Release of TV-46000, A Novel Long-Acting Injectable (LAI) Formulation of Risperidone, Under Extreme Conditions in Dogs, Minipigs and Humans
Source: Pharmaceutics. 2025 Jan 22;17(2):150. doi: 10.3390/pharmaceutics17020150 (PMC11859501; doi:10.3390/pharmaceutics17020150)
Supplement: Supplementary file 1 [file pharmaceutics-17-00150-s001.zip › pharmaceutics-3337740-supplementary.pdf]

pharmaceutics

MDPI

Supplementary Materials

An Evaluation of the Subcutaneous Depot Release of TV-46000, A Novel Long-Acting Injectable (LAI) Formulation of Risperidone, Under Extreme Conditions in Dogs, Minipigs and Humans

Lilach Steiner, David Bibi *, Avia Merenlender Wagner, Pavel Farkas, Safra Rudnick-Glick, Pippa Loupe and Hussein Hallak

Table S1. Mean PK parameters (±SD) following TV-46000 sc injections for TAM concentrations in male Beagle dogs following rubbing at the injection site at different timepoints vs non-rubbing controls.

	**Group 1 No rubbing (n = 6)**	**Group 2 0 h post-dose (n = 6)**	**Group 3 0.5 h post-dose (n = 6)**	**Group 4 1.0 h post-dose (n = 6)**	**Group 5 4.0 h post-dose (n = 6)**
C_{max} (ng/mL)	29.1 (1.4)	33.8 (1.3)	20.2 (1.3)	23.7 (1.6)	29.7 (1.2)
T_{max} (h)[a]	4 (3-8)	4 (2-8)	8 (3-120)	4 (3-168)	4 (3-8)
$T_{1/2}$ (h)	156.8 (42.9)	223.5 (122.5)	180.6 (49.5)	156 (26.2)	153 (44.5)
$AUC_{0\text{-tlast}}$ (ng × h/mL)	5600 (1.3)	7030 (1.3)	4710 (1.2)	3840 (1.4)	4750 (1.3)
$AUC_{0\text{-}\infty}$ (ng × h/mL)	5990 (1.3)	7260 (1.3)	5040 (1.2)	4100 (1.4)	5060 (1.4)

[a] Range (min-max range).
